# Supplementary material for: End Sequence Analysis Toolkit (ESAT) expands the extractable information from single-cell RNA-seq data
Source: Genome Res. 2016 Oct;26(10):1397–410. doi: 10.1101/gr.207902.116 (PMC5052061; doi:10.1101/gr.207902.116)
Supplement: Supplemental Material [file supp_gr.207902.116_Supp_Materials.tar.gz › ESAT-esat_v0.1/doc/html/classbroad_1_1core_1_1annotation_1_1_basic_genomic_annotation_reader.html]

ESAT: broad.core.annotation.BasicGenomicAnnotationReader Class Reference


ESAT


- Main Page
- Related Pages
- Packages
- Classes
- Files

- Class List
- Class Index
- Class Hierarchy
- Class Members

All Classes Namespaces Files Functions Variables Enumerator Pages

- broad
- core
- annotation
- BasicGenomicAnnotationReader

Public Member Functions |
Static Public Member Functions |
Static Public Attributes |
Protected Member Functions |
Protected Attributes |
List of all members

broad.core.annotation.BasicGenomicAnnotationReader Class Reference

Inheritance diagram for broad.core.annotation.BasicGenomicAnnotationReader:

[legend]

Collaboration diagram for broad.core.annotation.BasicGenomicAnnotationReader:

[legend]

|  |  |
| --- | --- |
| Public Member Functions | |
|  | BasicGenomicAnnotationReader () |
|  | |
|  | BasicGenomicAnnotationReader (String annotationFile) throws FileNotFoundException |
|  | |
|  | BasicGenomicAnnotationReader (String annotationFile, String annotationType) throws FileNotFoundException |
|  | |
| List< GenomicAnnotation > | getAnnotationList () |
|  | |
| List< GenomicAnnotation > | find (final String chr, final int startPos, final int endPos) throws IOException, ParseException |
|  | |
| void | load () throws IOException, ParseException |
|  | |
| void | plot (Graphics2D g2d, int start, int end, List< GenomicAnnotation > annots) |
|  | |
| GenomicAnnotation | createAnnotation (GenomicAnnotation a) |
|  | |
| int | parse (String file, GenomicAnnotationFilter< GenomicAnnotation > filter, AnnotationHandler handler) throws ParseException, IOException |
|  | |
| int | parse (BufferedReader br, GenomicAnnotationFilter< GenomicAnnotation > filter, AnnotationHandler handler) throws ParseException, IOException |
|  | |

|  |  |
| --- | --- |
| Static Public Member Functions | |
| static List< GenomicAnnotation > | parseRawRegions (String[] rawRegions) |
|  | |
| static List< GenomicAnnotation > | parseRawPointRegions (String[] rawRegions) |
|  | |

|  |  |
| --- | --- |
| Static Public Attributes | |
| static int | RIGHT\_MARGIN = 50 |
|  | |
| static int | VERTICAL\_SPACING = 100 |
|  | |
| static int | INTER\_VERTICAL\_SPACING = 40 |
|  | |
| static final Pattern | REG\_PATTERN = Pattern.compile("(chr)(\\w{1,2})(:)([0-9]+)(-)([0-9]+)") |
|  | |
| static final Pattern | POINT\_PATTERN = Pattern.compile("(chr)(\\w{1,2})(:)([0-9]+)") |
|  | |

|  |  |
| --- | --- |
| Protected Member Functions | |
| void | setAnnotationList (List< GenomicAnnotation > list) |
|  | |

|  |  |
| --- | --- |
| Protected Attributes | |
| File | source |
|  | |

## Constructor & Destructor Documentation

|  |  |  |  |  |
| --- | --- | --- | --- | --- |
| broad.core.annotation.BasicGenomicAnnotationReader.BasicGenomicAnnotationReader | ( |  | ) |  |

|  |  |  |  |  |  |
| --- | --- | --- | --- | --- | --- |
| broad.core.annotation.BasicGenomicAnnotationReader.BasicGenomicAnnotationReader | ( | String | *annotationFile* | ) | throws FileNotFoundException |

|  |  |  |  |
| --- | --- | --- | --- |
| broad.core.annotation.BasicGenomicAnnotationReader.BasicGenomicAnnotationReader | ( | String | *annotationFile*, |
|  |  | String | *annotationType* |
|  | ) |  | throws FileNotFoundException |

## Member Function Documentation

|  |  |  |  |  |  |
| --- | --- | --- | --- | --- | --- |
| GenomicAnnotation broad.core.annotation.BasicGenomicAnnotationReader.createAnnotation | ( | GenomicAnnotation | *a* | ) |  |

|  |  |  |  |
| --- | --- | --- | --- |
| List<GenomicAnnotation> broad.core.annotation.BasicGenomicAnnotationReader.find | ( | final String | *chr*, |
|  |  | final int | *startPos*, |
|  |  | final int | *endPos* |
|  | ) |  | throws IOException, ParseException |

Here is the call graph for this function:

|  |  |  |  |  |
| --- | --- | --- | --- | --- |
| List<GenomicAnnotation> broad.core.annotation.BasicGenomicAnnotationReader.getAnnotationList | ( |  | ) |  |

Here is the caller graph for this function:

|  |  |  |  |  |
| --- | --- | --- | --- | --- |
| void broad.core.annotation.BasicGenomicAnnotationReader.load | ( |  | ) | throws IOException, ParseException |

Here is the caller graph for this function:

|  |  |  |  |
| --- | --- | --- | --- |
| int broad.core.annotation.BasicGenomicAnnotationReader.parse | ( | String | *file*, |
|  |  | GenomicAnnotationFilter< GenomicAnnotation > | *filter*, |
|  |  | AnnotationHandler | *handler* |
|  | ) |  | throws ParseException, IOException |

|  |  |  |  |
| --- | --- | --- | --- |
| int broad.core.annotation.BasicGenomicAnnotationReader.parse | ( | BufferedReader | *br*, |
|  |  | GenomicAnnotationFilter< GenomicAnnotation > | *filter*, |
|  |  | AnnotationHandler | *handler* |
|  | ) |  | throws ParseException, IOException |

|  |  |  |  |  |  |
| --- | --- | --- | --- | --- | --- |
| static List<GenomicAnnotation> broad.core.annotation.BasicGenomicAnnotationReader.parseRawPointRegions | ( | String[] | *rawRegions* | ) |  |

static

|  |  |  |  |  |  |
| --- | --- | --- | --- | --- | --- |
| static List<GenomicAnnotation> broad.core.annotation.BasicGenomicAnnotationReader.parseRawRegions | ( | String[] | *rawRegions* | ) |  |

static

Parses an array of cannonical region descriptors of the form chrN:start-end

Parameters
:   |  |  |
    | --- | --- |
    | rawRegions |  |

Returns

|  |  |  |  |
| --- | --- | --- | --- |
| void broad.core.annotation.BasicGenomicAnnotationReader.plot | ( | Graphics2D | *g2d*, |
|  |  | int | *start*, |
|  |  | int | *end*, |
|  |  | List< GenomicAnnotation > | *annots* |
|  | ) |  |  |

Here is the call graph for this function:

|  |  |  |  |  |  |
| --- | --- | --- | --- | --- | --- |
| void broad.core.annotation.BasicGenomicAnnotationReader.setAnnotationList | ( | List< GenomicAnnotation > | *list* | ) |  |

protected

## Member Data Documentation

int broad.core.annotation.BasicGenomicAnnotationReader.INTER\_VERTICAL\_SPACING = 40

static

final Pattern broad.core.annotation.BasicGenomicAnnotationReader.POINT\_PATTERN = Pattern.compile("(chr)(\\w{1,2})(:)([0-9]+)")

static

final Pattern broad.core.annotation.BasicGenomicAnnotationReader.REG\_PATTERN = Pattern.compile("(chr)(\\w{1,2})(:)([0-9]+)(-)([0-9]+)")

static

int broad.core.annotation.BasicGenomicAnnotationReader.RIGHT\_MARGIN = 50

static

File broad.core.annotation.BasicGenomicAnnotationReader.source

protected

int broad.core.annotation.BasicGenomicAnnotationReader.VERTICAL\_SPACING = 100

static

---

The documentation for this class was generated from the following file:

- java/broad/core/annotation/BasicGenomicAnnotationReader.java


---

Generated on Fri Aug 15 2014 15:10:50 for ESAT by  

 1.8.7
